# Analysis of CNVs of *CFTR* gene in Chinese Han population with CBAVD

**DOI:** 10.1002/mgg3.1506

**Published:** 2020-09-19

**Authors:** Chengquan Ma, Ruyi Wang, Tengyan Li, Hongjun Li, Binbin Wang

**Affiliations:** ^1^ Department of Urology Peking Union Medical College Hospital Peking Union Medical College Chinese Academy of Medical Sciences Beijing P. R. China; ^2^ Graduate School of Peking Union Medical College Beijing P. R. China; ^3^ Center for Genetics National Research Institute for Family Planning Beijing P. R. China

**Keywords:** *CFTR*, Chinese Han population, Congenital bilateral absence of vas deferens, copy number variants

## Abstract

**Background:**

Congenital bilateral absence of vas deferens (CBAVD) is an important disease of male infertility, which affects 1%–2% of infertile population. In addition to common mutations of *CFTR*, copy number variants (CNVs) have also been implicated as one of the pathogenesis of CBAVD. The present study aimed to investigate the genetic contribution of *CFTR* CNVs in Chinese Han population with CBAVD.

**Methods:**

Two hundred and sixty‐three CBAVD patients were recruited. Genomic DNA was extracted from peripheral blood samples. The Multiplex Ligation‐dependent Probe Amplification assay was performed which targets the region of the *CFTR* gene.

**Results:**

Among 263 Chinese men affected with CBAVD in this study, 5 (1.90%) patients were detected for copy number variants in the region of *CFTR* gene (4 of them carried partial deletions and 1 of them carried partial duplication of *CFTR* gene).

**Conclusions:**

The study showed that the rate of *CFTR* CNVs in Chinese population with CBAVD were basically consistent with the previous reports. And the study first revealed genetic risk of CNVs of *CFTR* on a large sample size of CBAVD patients in Chinese Han population, which prompted that it was necessary to detect CNVs of *CFTR* in Chinese Han people with CBAVD.

## Graphical abstract

The study showed that the rate of CFTR CNVs in Chinese CBAVD population were basically consistent with the previous reports. And the study firstly revealed genetic risk of CNVs of CFTR on a large sample size of CBAVD patients in Chinese Han population, which prompted that it was necessary to detect CNVs of CFTR in Chinese Han people with CBAVD.

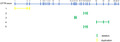

## 1 INTRODUCTION

Congenital bilateral absence of vas deferens (CBAVD) is an important disease of male infertility, accounting for 1%–2% of infertile population (Jequier, Ansell, & Bullimore, 1985; Yu, Chen, Ni, & Li, 2012). CBAVD is characterized by absence of vas deferens and semen abnormality (Practice Committee of the American Society for Reproductive Medicine in collaboration with the Society for Male & Urology, 2018).

Cystic fibrosis (CF) is a well‐known genetic disease, and showed racial differences (it is common in Caucasians but not common in Asians) (Yang, Wang, Zhang, Li, & Wang, 2018). Cystic fibrosis transmembrane conductance regulator gene (*CFTR*) (OMIM: 602421) defect can cause CF (Zielenski & Tsui, 1995), and Cystic Fibrosis Mutation Database (CFMDB) (https://www.genet.sickkids.on.ca/cftr/app) has been established.

Also, *CFTR* mutations were closely related to CBAVD (Cai et al., 2019; Chillón et al., 1995), which occurred in more than three quarters of CBAVD patients (Yu et al., 2012). In addition to common mutations (single nucleotide variants or small insertions and deletions), copy number variants (CNVs) (including large deletions and duplications) have also been implicated as one of the pathogenesis of CBAVD (Hantash et al., 2006; Ratbi et al., 2007; Taulan et al., 2007).

However, traditional methods cannot identify all *CFTR* variants (Férec et al., 2006; Hwang et al., 2018). Currently, Multiplex Ligation‐dependent Probe Amplification (MLPA) is an effective method to detect CNVs (Schrijver, Rappahahn, Pique, Kharrazi, & Wong, 2008; Taulan et al., 2012). Therefore, we used this technique to investigate the genetic contribution of *CFTR* CNVs in Chinese Han population with CBAVD.

## 2 MATERIALS AND METHODS

### 2.1 Ethical compliance

The study was approved by the Research Ethics Committee of Peking Union Medical College Hospital. And all participants signed informed consent forms.

### 2.2 Participants

From 2012 to 2018, 263 CBAVD patients were recruited from Urological Department of Peking Union Medical College Hospital. These patients are all Chinese Han people ages ranging from 21 to 49 years (29 ± 4.85). Patients claimed infertility after a few years of marriage. According to the guidelines published by the American Society for reproductive genetics in 2018 (Practice Committee of the American Society for Reproductive Medicine in collaboration with the Society for Male & Urology, 2018) and previous studies of our research group (Yang et al., 2018), inclusion criteria of CBAVD patients were as follows: impalpable vas deferens, absence of vas deferens by ultrasound, semen abnormality (volume <2.0 ml, pH value <7.2, and fructose <25 μM/ejaculate), normal serum follicle‐stimulating hormone levels, and luteinizing hormone levels, no typical symptoms of CF except for CBAVD.

### 2.3 DNA extraction

Genomic DNA from 263 CBAVD patients were extracted from peripheral venous blood using QIAamp DNA Blood Mini Kit (Qiagen, Hilden, Germany).

### 2.4 MLPA assay

The probes targeted the exon region of *CFTR* gene (Reference sequence numbers NM_000492.4). The PCR products were analyzed by capillary electrophoresis. The fluorescence data were processed by Applied Biosystems 3730xl DNA Analyzer, and the peak area data were collected. Relative peak area of *CFTR* probe recognition sequence was compared with the reference gene. Dosage quotient (DQ) was used as the judging basis of CNVs. We considered normal results with values between 0.8 and 1.2. DQ values equal to zero were considered homozygous deletion. DQ values between 0.4 and 0.65 were considered heterozygous deletion, DQ values between 1.3 and 1.65 were considered heterozygous duplication.

### 2.5 Evaluation of CNVs detected according to American College of Medical Genetics and Genomics (ACMG) guidelines

The CNV interpretation scoring metric put forward by ACMG was used to evaluate the impact on human health. Five aspects were studied: initial assessment of genomic content, overlap with established haploinsufficient regions, number of protein‐coding genes, evaluation of genomic content using public databases and literature, and evaluation of inheritance pattern for patient.

## 3 RESULTS

### 3.1 MLPA assay targeted *CFTR* gene on CBAVD patients

**FIGURE 1:**
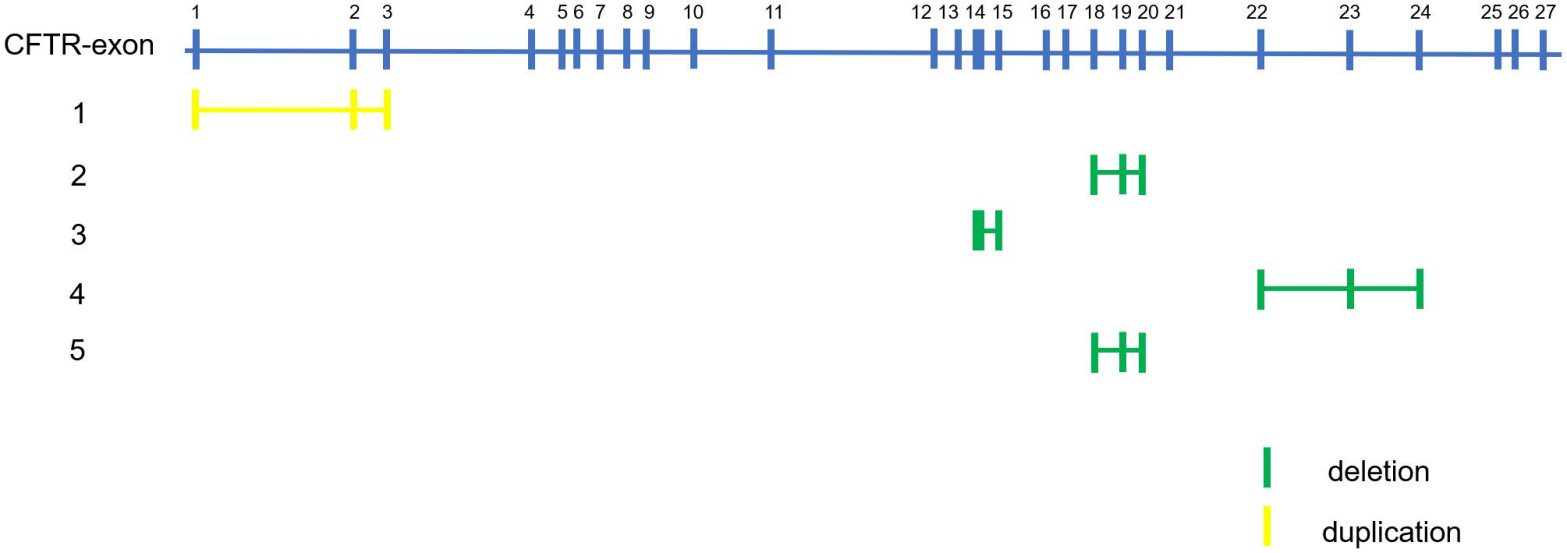
Distribution of copy number variants on CFTR exons. CFTR has 27 exons, 5 patients carried different CNVs. Yellow rectangles symbol duplication. Green rectangles symbol deletion

Among 263 Chinese men affected with CBAVD in this study, 5 (1.90%) patients were detected for copy number variants in the region of exons of the *CFTR* gene, including 1 patient carried heterozygous duplication of exons 1–3 of *CFTR* (*CFTR*dup1‐3, Legacy name), 2 patients carried heterozygous deletion of exons 18–20 of *CFTR*, 1 patient carried heterozygous deletion of exons 14–15 of *CFTR*, and 1 patient carried heterozygous deletion of exons 22–24 of *CFTR* (Table 1). In summary, four of them carried partial deletions and 1 of them carried partial duplication of *CFTR* gene (Figure 1).

**TABLE 1:** CNVs of *CFTR* gene and clinical features in CBAVD patients

Patient No.	Copy number status of *CFTR* ^a^	Dosage quotient	Age (years)	Left testicular volume (ml)	Right testicular volume (ml)	Left epididymis	Right epididymis	FSH	LH	PRL	E2	T
217	Heterozygous deletion of exons 14–15	0.64(E14); 0.65(E15)	31	13	13	Normal	Normal	2.14	0.98	5.2	61.8	3
243	Heterozygous deletion of exons 18–20	0.45(E18); 0.5(E19); 0.47(E20)	33	13	13	Normal	Normal	5.1	3.96	16.3	40.7	3.42
299	Heterozygous duplication of exons 1–3	1.65(E1); 1.33(E2); 1.41(E3)	31	10	10	Normal	Normal	3.4	5.95	10	70.7	8.39
324	Heterozygous deletion of exons 22–24	0.45(E22); 0.44(E23); 0.57(E24)	28	10	10	Normal	Normal	4.4	3.65	6.52	20.8	3.93
357	Heterozygous deletion of exons 18–20	0.45(E18); 0.57(E19); 0.47(E20)	27	15	15	Normal	Normal	2.83	2.19	3.59	73.4	2.94

Note:

Testicular volume (normal range: 15–25 ml) FSH, Follicle stimulating hormone (normal range: 1.27–12.96 mIU/mL); LH, Luteinizing hormone (normal range: 1.24–8.62 mIU/mL); PRL, Prolactin (normal range: 2.64–13.13 ng/ml); E2, Estradiol (normal range: 20–75 pg/mL);T, Testosterone (normal range: 4–8 ng/mL).

^a^ *CFTR*: Reference sequence numbers NM_000492.4.

### 3.2 Clinical characteristics of five patients with CNVs

The five patients met the inclusion criteria described above. They have impalpable vas deferens but normal testis and epididymis. Their hormone levels are basically normal. See Table 1 for details.

### 3.3 The impact of CNVs on human health

Three CNVs (*CFTR*dup1‐3, deletion of exons 22–24 and deletion of exons 18–20) have been reported. Only deletion of exons 14–15 of *CFTR* was not reported before. Three CNVs (deletion of exons 14–15, deletion of exons 22–24 and deletion of exons 18–20) were evaluated to be pathogenic. *CFTR*dup1‐3 was evaluated to be uncertain significance. See details in Table 2.

**TABLE 2:** CNV Interpretation Scoring Metric of ACMG

Patient No.	Copy number status	Contain protein‐coding elements (Score)	Overlap with established haploinsufficient genes (Score)^a^	Number of protein‐coding RefSeq genes (Score)	Analysis of Public Databases and Literature	Inheritance Pattern for Patient (Score)	Total score	Classification^c^
217	Heterozygous deletion of exons 14–15	Yes (0)	Both breakpoints are within *CFTR* and the exons are in biologically‐relevant transtripts (0.9)	1 (0)	Skip	Inheritance information is unavailable but the patient has highly specific phenotype of CBAVD(0.3)	1.2	Pathogenic
243	Heterozygous deletion of exons 18–20	Yes (0)	Both breakpoints are within *CFTR* and the exons are in biologically‐relevant transtripts (0.9)	1 (0)	Skip	Inheritance information is unavailable but the patient has highly specific phenotype of CBAVD(0.3)	1.2	Pathogenic
299	Heterozygous duplication of exons 1–3	Yes (0)	Both breakpoints are within *CFTR* and the type is presumed in tandem duplication (0.45)	1 (0)	The patient has a highly specific phenotype consistent with *CFTR*, but the inheritance is unknown (0.1). And the CNV did not overlap with common population variation^b^	Inheritance information is unavailable but the patient has highly specific phenotype of CBAVD(0.3)	0.85	Uncertain Significance
324	Heterozygous deletion of exons 22–24	Yes (0)	Both breakpoints are within *CFTR* and the exons are in biologically relevant transtripts (0.9)	1 (0)	Skip	Inheritance information is unavailable but the patient has highly specific phenotype of CBAVD(0.3)	1.2	Pathogenic
357	Heterozygous deletion of exons 18–20	Yes (0)	Both breakpoints are within *CFTR* and the exons are in biologically relevant transtripts (0.9)	1 (0)	Skip	Inheritance information is unavailable but the patient has highly specific phenotype of CBAVD(0.3)	1.2	Pathogenic

^a^ The haploinsufficiency evaluation of CFTR has been established (https://dosage.clinicalgenome.org/).

^b^ The Heterozygous duplication of exons 1–3 did not overlap with common population variation (http://dgv.tcag.ca/).

^c^ Pathogenic: 0.99 or more points; Likely Pathogenic: 0.90 to 0.98; Variant of Uncertain Significance: 0.89 to −0.89; Likely Benign: −0.90 to −0.98; Benign: −0.99 or fewer points.

## 4 DISCUSSION

In the Cystic Fibrosis Mutation Database (www.genet.sickkids.on.ca/) and reports in other database, most of the results we found has been detected. *CFTR*dup1‐3 was evaluated to be uncertain significance, but T. Casals et al identified *CFTR*dup1‐3 from two unrelated Spanish CF alleles (Ramos, Gartner, & Casals, 2008). While Hantash FM et al identified the deletion of exons 22–24 of *CFTR* in a CBAVD patient (Hantash et al., 2006). Chevalier‐Porst and Bozon (2000) identified the deletion of exons 18–20 in a patient who has sweat chloride 82 mmol/l.

And we found a CNV (deletion of exons 14–15 of *CFTR*) from a CBAVD patients which was not reported before. No identical overlapped variant was found in Database of Genomic Variants (DGV, http://dgv.tcag.ca/dgv/app/home/) and Genome Aggregation Database (GnomAD, http://gnomad.broadinstitute.org/). Thus, this heterozygous deletion of *CFTR* was considered rare in the general population. CFTR consists of five domains, including two transmembrane‐spanning domains (MSD1 and MSD2), two nucleotide‐binding domains (NBDs) and a regulatory domain (R) (Morales et al., 1996). Exon 14 and exon 15 was located in the regulatory domain and MSD2. 2 MSDs form selective chloride channels, while phosphorylation of R controls channel activity (Sheppard & Welsh, 1999). Deletion of exons 14–15 may be destructive to the function of the CFTR protein. According to the American College of Medical Genetics and Genomics guidelines (Riggs et al., 2020), we thought that deletion of exons 14–15 of *CFTR* maybe pathogenic (see details in Table 2).

Almost all men with CF have the symptoms of CBAVD (Practice Committee of the American Society for Reproductive Medicine in collaboration with the Society for Male & Urology, 2018), and assessment of patients with CNVs (large deletions and duplications) in our study showed that they have CBAVD typical clinical characteristics. Furthermore, patients with large deletions had more severe outcomes due to reduced function of the protein (Hantash et al., 2006; Taulan et al., 2007). While large duplications also contributed to morbidity (Costantino et al., 2011; Martins et al., 2019; Paracchini et al., 2008; Petrova et al., 2019). So the CNVs found in our study contributed to CBAVD genetically.

CNVs have occurred in both CF and CBAVD. In the world, there are 2% of large deletions or duplications of *CFTR* which affect from single exons to entire gene (Taulan et al., 2012). In a study of Brazil population, 3 (1.82%) CNVs (*CFTR*dup2‐3, *CFTR*del25‐26, and *CFTR*del25‐27‐CTTNBP2) were identified in 165 CF patients (Martins et al., 2019). In a study of Ecuadorian population, five (3.55%) cases were identified with three deletions (deletion of *CFTR* exon 10, deletion of *CFTR* exon 12, *CFTR*dele22,23) in 141 CF patients using microarrays (Ruiz‐Cabezas et al., 2019). large genomic deletions of *CFTR* (*CFTR*dele2 and *CFTR*dele22_24) was identified in 2 (1.10%) of the 182 samples affected with CBAVD analyzed in a study of European population (Taulan et al., 2007).

Among 263 Chinese men affected with CBAVD in our study, 5 (1.90%) patients were detected for CNVs in the region of exons of the *CFTR* gene. Refer to previous studies, four (1.80%) large rearrangements (two partial deletions (exons 17a–18 and 22–23), a complete deletion and a partial duplication (exons 11–13)) of *CFTR* were found in 222 CBAVD patients in a French study using semi‐quantitative fluorescent multiplex PCR (QFM‐PCR) assay (Ratbi et al., 2007). A large deletion of *CFTR* (deletion of exons 22–24) in one (2.08%) of the 48 CBAVD patients was identified in an American study (Hantash et al., 2006). Detection rate of CNVs basically consistent with previous studies suggested that the use of the MLPA technique in our study was effective for the detection of CNVs. Given to the research mentioned above, the results showed less obvious racial differences in CNVs of *CFTR* in different CBAVD populations.

However, Yuan et al. in 2019 showed that two *CFTR* gene mutations in 58.33% (42/72) of Congenital absence of vas deferens (CAVD) patients, only one mutation in 18.06% (13/72) and no mutation in 23.61% (17/72). And they did not find deletions or duplications using MLPA analysis on *CFTR* gene in 30 CAVD patients (with one/no mutation) (Yuan et al., 2019). In 2020, Wang et al. found 15 *CFTR* mutations in 15 CBAVD patients among 38 samples (Wang et al., 2020), but they did not detect CNVs. CNV screening was performed on some samples of CAVD in these two studies, but no CNV was found. Our study was conducted in a large sample of cases, but CNV was found only in 1.9% of the samples. As there are less CNV in CBAVD, CNV may not be found in small samples. And to our best knowledge, this study first reported CNVs of *CFTR* on a large sample size of CBAVD patients in Chinese population.

Our results also showed that the detection of CNVs of *CFTR* would be significant for genetic counseling in Chinese Han population with CBAVD. Some mutations may exist in patients without *CFTR* abnormalities using traditional detection methods. Traditional testing also means comprehensive screening, such as F508del and 100‐mutation panels. However 10%–40% of the mutations cannot be identified by common methods (Practice Committee of the American Society for Reproductive Medicine in collaboration with the Society for Male & Urology, 2018). Before the treatment of assisted reproduction, genetic factors of the patients with normal spermatogenesis should be analyzed, so as to provide better reproductive guidance (Meng et al., 2001). But given to relatively low positive rate (1.90% in our study, no *CFTR* CNVs found in other Chinese studies (Wang et al., 2020; Yuan et al., 2019) and the conditions for detecting CNVs in previous studies (Hantash et al., 2006; Ratbi et al., 2007; Taulan et al., 2007), we recommended that CNV screening should be performed in CBAVD patients with one/no mutation.

It is worth noting that adhesion G‐protein coupled receptor G2 (*ADGRG2*) gene (OMIM: 300572) was also related to CBAVD with X‐linked inheritance pattern (Patat et al., 2016). Yuan et al. in 2019 identified 2 novel *ADGRG2* mutations in 10 CBAVD patients without *CFTR* mutations (Yuan et al., 2019) and Wang et al. identified one *ADGRG2* mutations in 38 CBAVD patients (Wang et al., 2020) in Chinese populations. Also, other genes may also be associated with CBAVD, for example, *SLC9A3* (OMIM: 182307) deletion occurred in some Taiwanese CBAVD patients without common *CFTR* mutations (Wang et al., 2017).

In our study, we found that it was feasible to use the MLPA assay to test the CNVs (large deletions and duplications) of *CFTR* in our patients. Our results showed that CNVs of *CFTR* contributed to CBAVD genetically. And 1.90% CBAVD patients carried CNVs of exons of *CFTR* in Chinese Han populations, which was basically consistent with the previous reports. While this study first revealed genetic risk of CNVs of *CFTR* on a large sample size of CBAVD patients in Chinese Han population, and prompted that it was necessary to detect CNVs of *CFTR* in Chinese Han people with CBAVD.

## 5 CONCLUSIONS

The study showed that 1.90% (5/263) CBAVD patients carried CNVs of exons of *CFTR* in Chinese Han populations by MLPA, suggesting that the rate of *CFTR* CNVs in Chinese CBAVD population were basically consistent with the previous reports. And this study first revealed genetic risk of CNVs of *CFTR* on a large sample size of CBAVD patients in Chinese Han population, which prompted that it was necessary to detect CNVs of *CFTR* in Chinese Han people with CBAVD.

## CONFLICT OF INTEREST

The authors declare that they have no competing interests.

## AUTHORS’ CONTRIBUTIONS

All authors contributed to the study conception and design. Material preparation were performed by Chengquan Ma and Tengyan Li, data collection and analysis were performed by Binbin Wang, Hongjun Li, and Jing Wang. The first draft of the manuscript was written by Chengquan Ma and Ruyi Wang, and all authors commented on previous versions of the manuscript. All authors read and approved the final manuscript.
